# Supplementary material for: Assessing food safety practices and foodborne illness risk factors in Brazilian households
Source: PLoS One. 2025 Jun 18;20(6):e0325070. doi: 10.1371/journal.pone.0325070 (PMC12176236; doi:10.1371/journal.pone.0325070)
Supplement: S1 Table — (DOCX) [file pone.0325070.s001.docx]

| **Questions**  **Table S1: Full Questionnaire Responses: Distribution of Answer Counts and Percentages Across Clusters and Total Respondents.** | **Category** | **ALL (n = 1,043)** | **Cluster1 (n = 136)** | **Cluster 2 (n = 88)** | **Cluster 3 (n = 464)** | **Cluster 4 (n = 355)** |
| --- | --- | --- | --- | --- | --- | --- |
| Do you wash your hands before ingesting food? | No | 22 (2.11%) | 1 (0.74%) | 1 (1.14%) | 17 (3.66%) | 3 (0.84%) |
|  | Yes, but rarely | 49 (4.69%) | 4 (2.94%) | 2 (2.27%) | 38 (8.19%) | 5 (1.40%) |
|  | Yes, sometimes | 494 (47.32%) | 49 (36.03%) | 41 (46.59%) | 258 (55.60%) | 146 (41.01%) |
|  | Yes, always | 478 (45.88%) | 82 (60.29%) | 44 (50.00%) | 151 (32.54%) | 201 (56.74%) |
| Do you wash your hands before preparing food at home? | No | 9 (0.86%) | 0 | 2 (2.27%) | 6 (1.29%) | 1 (0.28%) |
|  | Yes, but rarely | 11 (1.05%) | 0 | 1 (1.14%) | 9 (1.94%) | 1 (0.28%) |
|  | Yes, sometimes | 136 (13.03) | 9 (6.62%) | 11 (12.50%) | 92 (19.83%) | 24 (6.74%) |
|  | Yes, always | 887 (85.06%) | 127 (93.38%) | 74 (84.09%) | 357 (76.94%) | 329 (92.70%) |
| Do you wash your vegetables before eating? | No | 13 (1.25%) | 0 | 0 | 9 (1.94%) | 4 (1.12%) |
|  | Yes, with water | 549 (53.68%) | 74 (54.41%) | 52 (59.09%) | 281 (60.56%) | 142 (40.17%) |
|  | Yes, with water and commercial vegetable disinfectant | 166 (15.90%) | 33 (24.26%) | 14 (15.91%) | 59 (12.72%) | 60 (16.85%) |
|  | Yes, with water and sodium hypochlorite | 315 (30.17%) | 29 (21.32%) | 22 (25.00%) | 115 (24.78%) | 149 (41.85%) |
| Do you use one dishcloth for utensils and dishes and another one for hands? | No | 531 (50.96%) | 52 (38.24%) | 48 (54.55%) | 274 (59.05%) | 157 (44.38%) |
| With what frequency do you wash or change the dishcloths? | Every two months or less | 15 (1.44%) | 0 | 5 (5.68%) | 7 (1.51%) | 3 (0.84%) |
|  | Monthly | 34 (3.26%) | 2 (1.47%) | 8 (9.09%) | 20 (4.31%) | 4 (1.12%) |
|  | Twice a month | 117 (11.21) | 7 (5.15%) | 9 (10.23%) | 78 (16.81%) | 23 (6.46%) |
|  | Weakly or more | 877 (84.10%) | 127 (93.38%) | 66 (75.00%) | 359 (77.37%) | 325 (91.57%) |
| With what frequency do you wash/disinfect your sink? | Monthly | 61 (5.84%) | 1 (0.74%) | 12 (13.64%) | 41 (9.27%) | 5 (1.40%) |
|  | Weakly | 280 (26.82%) | 17 (12.50%) | 32 (36.36%) | 162 (34.91%) | 69 (19.38%) |
|  | Daily | 423 (40.52%) | 65 (47.79%) | 31 (35.23%) | 161 (34.70%) | 166 (46.63%) |
|  | After every food preparation | 279 (26.82%) | 53 (38.97%) | 13 (14.77%) | 98 (21.12%) | 115 (32.58%) |
| Do you mix different cleaning products/disinfectants to clean the kitchen? | No | 663 (63.60%) | 61 (44.85%) | 53 (60.23%) | 324 (69.83%) | 225 (63.48%) |
| With what frequency do you change the kitchen sponge | Monthly | 344 (32.95%) | 32 (23.53%) | 36 (40.91%) | 198 (42.67%) | 78 (21.91%) |
|  | Biweekly | 448 (42.91%) | 62 (45.59%) | 36 (40.91%) | 194 (41.81%) | 156 (43.82%) |
|  | Weakly | 248 (23.85%) | 39 (28.68%) | 16 (18.18%) | 72 (15.52%) | 121 (34.27%) |
|  | Daily | 3 (0.29%) | 3 (2.21%) | 0 | 0 | 0 |
| With what frequency do you clean the dish drainer? | Monthly or less | 581 (55.65%) | 40 (29.41%) | 68 (77.27%) | 337(72.64%) | 136 (38.20%) |
|  | Twice a month | 154 (14.75%) | 25 (18.38%) | 12 (13.64%) | 48 (10.34%) | 69 (19.38%) |
|  | Weakly | 227 (21.84%) | 51 (37.40%) | 5 (5.68%) | 57 (12.28%) | 115 (32.30%) |
|  | Daily | 81 (7.76%) | 20 (14.71%) | 3 (3.41%) | 22 (4.74%) | 36 (10.11%) |
| Do you use the same cutting board for meat and vegetables? | No | 366 (35.15%) | 52 (38.24%) | 21 (23.86%) | 143 (30.82%) | 151 (42.42%) |
| After using the cutting board or any utensils to prepare or cut raw meat, is this object washed before being reutilized? | No | 80 (7.66%) | 5 (3.68%) | 5 (5.68%) | 56 (12.07%) | 14 (3.93%) |
| Do you wash any meat before cooking? | No | 811 (77.78%) | 0 | 0 | 460 (99.14%) | 351 (98.88%) |
|  | Yes, pork | 3 (0.29%) | 0 | 0 | 1 (0.22%) | 2 (0.56%) |
|  | Yes, beef | 9 (0.86%) | 4 (2.94%) | 1 (1.14%) | 3 (0.65%) | 1 (0.28%) |
|  | Yes, chicken | 109 (10.44%) | 66 (48.53%) | 42 (47.73%) | 0 | 1 (0.28%) |
|  | Yes, all of them | 111 (10.63%) | 66 (48.53%) | 45 (51.14%) | 0 | 0 |
| Do you consume undercooked meat? | No | 522 (50.10%) | 85 (62.50%) | 50 (56.82%) | 224 (48.28%) | 163 (46.07%) |
| Do you use a thermometer to check the doneness of meat? | No | 1,011 (96.93%) | 134 (98.53%) | 86 (97.73%) | 448 (96.55%) | 343 (96.63%) |
| Do you eat raw meat, such as sashimi, ceviche, raw kibbeh and similar? | No | 411 (39.46%) | 77 (56.62%) | 37 (42.05%) | 166 (35.78%) | 131 (37.08%) |
| How do you defrost meat that is in the freezer to be prepared for the next day? | I don’t leave meat in the freezer | 17 (1.63%) | 0 | 1 (1.14%) | 14 (3.02%) | 2 (0.56%) |
|  | Put in the microwave | 109 (10.44%) | 15 (11.03%) | 6 (6.82%) | 57 (12.28%) | 31 (8.71%) |
|  | Leave on top of the kitchen countertop | 323 (30.94%) | 53 (38.97%) | 34 (38.64%) | 153 (32.97%) | 83 (23.31%) |
|  | Put in the refrigerator until it’s time to prepare | 594 (56.99%) | 68 (50.00%) | 47 (53.41%) | 240 (51.72%) | 239 (67.42%) |
| Do you store eggs inside the refrigerator? | No | 111 (10.73%) | 23 (16.91%) | 4 (4.55%) | 50 (10.78%) | 34 (9.83%) |
|  | Yes, in the refrigerator door | 260 (24.90%) | 54 (39.71%) | 14 (15.91%) | 129 (27.80%) | 63 (17.70%) |
|  | Yes, anywhere inside the refrigerator | 168 (16.09%) | 8 (5.88%) | 17 (19.32%) | 97 (20.91%) | 46 (12.92%) |
|  | Yes, in a specific compartment inside the refrigerator | 504 (48.28%) | 51 (37.50%) | 53 (60.23%) | 188 (40.52%) | 212 (59.55%) |
| Do you wash eggs before storing? | No | 960 (92.05%) | 23 (16.91%) | 4 (4.55%) | 50 (10.78%) | 34 (9.83%) |
|  | Yes, with water | 36 (3.45%) | 54 (39.71%) | 14 (15.91%) | 129 (27.80%) | 63 (17.70%) |
|  | Yes, scrubbing with sponge and water | 15 (1.44%) | 8 (5.88%) | 17 (19.32%) | 97 (20.91%) | 46 (12.92%) |
|  | Yes, scrubbing with sponge, water and detergent | 32 (3.07%) | 51 (37.50%) | 53 (60.23%) | 188 (40.52%) | 212 (59.55%) |
| Do you consume raw or soft yolk eggs? | No | 605 (58.05%) | 90 (66.18%) | 54 (61.36%) | 255 (54.96%) | 206 (58.15%) |
| How do you store leftovers for the next day? | I don’t eat leftovers | 11 (1.05%) | 0 | 1 (1.14%) | 5 (1.08%) | 5 (1.40%) |
|  | On the kitchen counter in a closed recipient | 14 (1.34%) | 1 (0.74%) | 3 (3.41%) | 6 (1.29%) | 4 (1.12%) |
|  | Inside the stove | 5 (0.48%) | 2 (1.47%) | 0 | 2 (0.43%) | 1 (0.28%) |
|  | Refrigerator | 998 (95.69%) | 126 (92.65%) | 83 (94.32%) | 449 (96.77%) | 340 (95.79%) |
|  | Freezer | 15 (1.44%) | 7 (5.15%) | 1 (1.14%) | 2 (0.43%) | 5 (1.40%) |
| With what frequency do you clean the silverware drawers? | Annually or less | 177 (16.95%) | 0 | 28 (31.82%) | 149 (32.11%) | 0 |
|  | Every six months | 152 (14.56%) | 3 (2.21%) | 16 (18.18%) | 128 (27.59%) | 5 (1.40%) |
|  | Every two-three months | 274 (26.25%) | 23 (16.91%) | 32 (36.26%) | 140 (30.17%) | 79 (22.19%) |
|  | Monthly | 249 (23.85%) | 50 (26.76%) | 12 (13.64%) | 41 (8.84%) | 146 (41.01%) |
|  | Twice a month | 113 (10.82%) | 28 (20.59%) | 0 | 5 (1.08%) | 80 (22.47%) |
|  | Weakly or more | 78 (7.57%) | 32 (23.53%) | 0 | 1 (0.22%) | 45 (12.92%) |
| With what frequency do you thoroughly clean the refrigerator? | Annually or less | 74 (7.09%) | 0 | 8 (9.09%) | 65 (14.01%) | 1 (0.28%) |
|  | Twice a year | 186 (17.82%) | 5 (3.68%) | 25 (28.41%) | 134 (28.88%) | 22 (6.18%) |
|  | Once every three months | 386 (36.97%) | 44 (32.35%) | 43 (48.86%) | 187 (40.30%) | 112 (31.46%) |
|  | Monthly or more | 398 (38.12%) | 87 (63.97%) | 12 (13.64%) | 78 (16.81%) | 220 (62.08%) |
| Do you separate different types of foods into different areas of the refrigerator? | No | 471 (45.11%) | 55 (40.44%) | 45 (51.14%) | 238 (51.29%) | 133 (37.36%) |
| Do you wash products bought at supermarkets before putting them on the fridge? | No | 703 (67.34%) | 72 (52.94%) | 51 (57.95%) | 359 (77.37%) | 221 (62.08%) |
| What is your average transport time of frozen goods from supermarket to freezer? | Thirty minutes | 786 (75.29%) | 98 (72.06%) | 56 (63.64%) | 351 (75.65%) | 281 (78.93%) |
|  | One to two hours | 246 (23.66%) | 36 (26.47%) | 32 (36.36%) | 105 (22.63%) | 74 (20.79%) |
|  | Two to four hours | 11 (1.05%) | 2 (1.47%) | 0 | 8 (1.71%) | 1 (0.28%) |
| Do you wait for the dish to cool down before refrigerating it? | No | 311 (29.79%) | 33 (24.26%) | 12 (13.64%) | 128 (27.59%) | 138 (38.76%) |
| Do you check the temperature of your refrigerator? | No | 660 (63.22%) | 62 (45.59%) | 71 (80.68%) | 323 (69.61%) | 204 (57.30%) |
| Do you use trash bins on your kitchen countertop? | No | 559 (53.64%) | 57 (41.91%) | 54 (61.36%) | 246 (53.02%) | 202 (57.02%) |
| Do you use a broom to sweep the kitchen floor? | No | 265 (25.38%) | 16 (11.76%) | 18 (20.45%) | 134 (28.88%) | 97 (27.25%) |
| Do you consume food past their expiration date? | No | 737 (70.69%) | 116 (85.29%) | 64 (72.73%) | 274 (59.05%) | 284 (79.78%) |
| Do you consume food past their secondary shelf life? | No | 524 (50.29%) | 91 (66.91%) | 45 (51.14%) | 177 (38.15%) | 211 (59.55%) |
| Do you remove the moldy part of food to eat the rest that doesn’t seen spoiled? | No | 905 (86.78%) | 123 (90.44%) | 71 (80.68%) | 390 (84.05%) | 322 (90.45%) |
| What is the origin of water you use as an ingredient for broths or soups? | Tap water | 429 (41.09%) | 49 (36.03%) | 43 (48.86%) | 239 (51.51%) | 98 (27.53%) |
|  | Gallon of mineral water | 56 (5.46%) | 11 (8.09%) | 7 (7.95%) | 16 (3.45%) | 22 (6.46%) |
|  | Filtered water | 558 (53.45%) | 76 (55.88%) | 38 (43.18%) | 209 (45.04%) | 235 (66.01%) |
