# Supplementary figures and images for: Assessing food safety practices and foodborne illness risk factors in Brazilian households
Source: PLoS One. 2025 Jun 18;20(6):e0325070. doi: 10.1371/journal.pone.0325070 (PMC12176236; doi:10.1371/journal.pone.0325070)

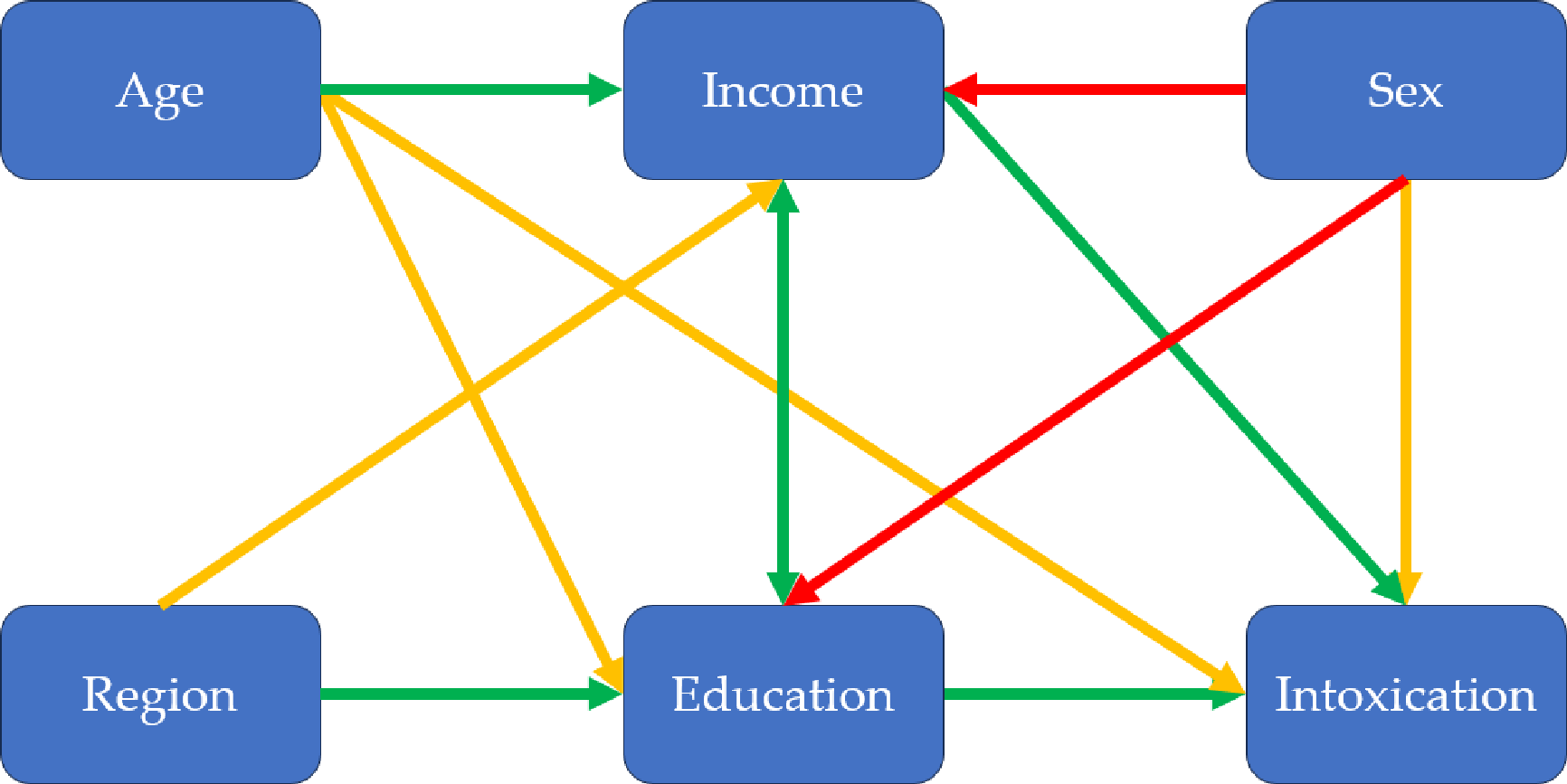

Supplement: S1 Fig — Causal diagram depicting the relationship between respondents’ profile and foodborne illness. The arrows represent the direction and relative strength of influence, categorized as follows: red arrows indicate the weakest influence, yellow arrows represent moderate influence, and green arrows signify the strongest influence. (TIF) [file pone.0325070.s002.tif]
