# Supplementary material for: Assessing food safety practices and foodborne illness risk factors in Brazilian households
Source: PLoS One. 2025 Jun 18;20(6):e0325070. doi: 10.1371/journal.pone.0325070 (PMC12176236; doi:10.1371/journal.pone.0325070)
Supplement: Attachment — Submitted filename: Response to Reviewers.docx [file pone.0325070.s005.docx]

Please note: All references to page and line numbers for requested changes are presented in the format (Page-Line), using the “Track Changes” version. **All changes related to this review are highlighted in yellow in the revised version.**

Academic editor:

1. Please ensure that your manuscript meets PLOS ONE's style requirements, including those for file naming.

**Response: Thank you for bringing to our attention the deviations from PLOS ONE’s formatting guidelines. We have revised the manuscript, including the authors' names and affiliations, to fully comply with the journal’s style requirements.**

2. You indicated that you had ethical approval for your study. In your Methods section, please ensure you have also stated whether you obtained consent from parents or guardians of the minors included in the study or whether the research ethics committee or IRB specifically waived the need for their consent.

**Response: We appreciate the suggestion. A clarifying sentence has been added to the Materials and Methods section (P2–79) stating that no minors were included in this study. Line 89 confirms a participant that stated being <18 years old was excluded.**

3. Please state what role the funders took in the study.  If the funders had no role, please state: "The funders had no role in study design, data collection and analysis, decision to publish, or preparation of the manuscript."

Please include this amended Role of Funder statement in your cover letter; we will change the online submission form on your behalf.

**Response: As our funders had no role in the study, we have added the following statement to the cover letter as requested: "The funders had no role in study design, data collection and analysis, decision to publish, or preparation of the manuscript."**

4. We note that you have indicated that there are restrictions to data sharing for this study. PLOS only allows data to be available upon request if there are legal or ethical restrictions on sharing data publicly.

a) If there are ethical or legal restrictions on sharing a de-identified data set, please explain them in detail (e.g., data contain potentially identifying or sensitive patient information, data are owned by a third-party organization, etc.) and who has imposed them (e.g., a Research Ethics Committee or Institutional Review Board, etc.). Please also provide contact information for a data access committee, ethics committee, or other institutional body to which data requests may be sent.

b) If there are no restrictions, please upload the minimal anonymized data set necessary to replicate your study findings to a stable, public repository and provide us with the relevant URLs, DOIs, or accession numbers. For a list of recommended repositories, please see

<https://journals.plos.org/plosone/s/recommended-repositories>. You also have the option of uploading the data as Supporting Information files, but we would recommend depositing data directly to a data repository if possible.

We will update your Data Availability statement on your behalf to reflect the information you provide.

**Response: Thank you for your inquiry. We have added an Excel file to the Supporting Information section (P15-690) with all the information collected from the questionnaire, with the exception of the e-mails and names of the respondents.**

Reviewer #1:

The manuscript presents a technically sound study investigating behavioral patterns related to food safety and their association with self-reported foodborne illness. The study is well-structured, employs rigorous statistical methods, and provides valuable insights into food safety behaviors. However, certain limitations, particularly in data availability and generalizability, should be addressed.
The study's methodology is appropriate, and statistical analyses have been conducted rigorously. The use of logistic regression, Spearman correlation, and cluster analysis is suitable for identifying behavioral patterns and risk factors. Ethical approval has been obtained, ensuring adherence to research guidelines. However, the reliance on self-reported foodborne illness cases introduces potential recall bias and misclassification risks. Additionally, the study sample is not fully representative, which may affect the broader applicability of findings.

**Response:** **We sincerely thank Reviewer 1 for the thoughtful and detailed feedback, which has greatly contributed to the improvement of our manuscript. The opportunity to refine our work and address these points is highly appreciated. All the alterations related to your review are highlighted in the revised manuscript (P9-373,380).**

Acknowledge the potential impact of recall bias more explicitly in the discussion.

**Response: A phrase has been added to the end of the Discussion section (P9-388) to more explicitly acknowledge the impact of potential recall bias on the findings.**

If possible, provide additional justification for the representativeness of the sample or discuss how future studies could address this limitation.

**Response: A sentence has been added to a paragraph of the Discussion section (P9–373) to suggest how future studies could address this limitation. This issue is further emphasized in the next paragraph (P9–380) to reinforce its potential impact on the generalizability of the findings.**

The conclusions align with the study's results, emphasizing the need for food safety education and intervention programs. However, due to the reliance on self-reported data, a causal relationship between behaviors and foodborne illness cannot be definitively established. The authors correctly acknowledge this limitation, but it should be further emphasized in the discussion.

**Response: To reinforce the observational nature of the study, we added two clarifying sentences at the end of the Discussion (P9–388) highlighting that the associations identified do not imply causation.**

Consider softening causal language in the conclusion to reflect the observational nature of the study.

**Response: We revised multiple sentences in the Conclusion section (P10–401, P10–402, P10–415) to soften causal language and better reflect the study’s cross-sectional design.**

Highlight future research directions, such as using objective, clinically/laboratory-confirmed cases to validate findings.

**Response: We have added two sentences in the Discussion (P9–368) to suggest future directions, including the use of laboratory-confirmed cases and prospective study designs.**

Provide a publicly accessible dataset containing anonymized individual data points or summary statistics.

**Response: This recommendation was also given by the academic editor. An Excel file with all the information gathered from our questionnaire was added to the Supporting Information section (P15-690).**

Reviewer #2:

This study provides valuable insight into food handling behaviors in Brazilian households and their potential link to self-reported foodborne illness.
The cross-sectional nature of the study limits its ability to establish causality. While it identifies associations between behaviors and self-reported illness, it cannot definitively claim that poor food handling caused these illnesses.

**Response: We thank the reviewer for this important observation. We fully agree that, as a cross‑sectional survey, our study cannot prove causality, but rather uncovers associations that can inform future work. To make this clear to readers, we have (following Reviewer #1’s recommendation) added phrases to the manuscript (P9-388). Despite this inherent limitation, we believe our large, diverse sample and robust cluster‑and‑risk‑factor analyses provide valuable insights into household food safety patterns in Brazil. By documenting these associations, our work lays the groundwork for hypothesis‑driven prospective or interventional studies, ultimately helping to prioritize the behaviors most in need of targeted educational campaigns.**

Reviewer #3:

While the authors indicate that data underlining the findings will be made available and provide a general availability statement, they do not specify the means by which this data can be accessed, which may limit transparency.

**Response: We thank the reviewer for highlighting this important point. In response, we have uploaded an Excel file to the Supporting Information section (P15–690) containing the anonymized data collected through the questionnaire, with all personally identifiable information removed.**

Note: Please attend to the error in the range given on page 5, line 186.

**Response: We do not believe there is an error on the range of this category. If the reviewer looks at Table S1:**

- **48.28% of respondents from cluster 3 said ‘no’ to consuming undercooked meat – meaning 51.72% said ‘yes’**
- **For the other clusters (1, 2 and 4), the % saying ‘no’ for this question varied from 46.07% to 62.50% for this question which means 53.93% – 37.5% responded they ate undercooked meat.**

**We hope that these revisions address all concerns raised and further improve the clarity and quality of the manuscript. Thank you for your valuable feedback and the opportunity to enhance this work.**
